# Seasonal morphotypes of *Drosophila suzukii* differ in key life‐history traits during and after a prolonged period of cold exposure

**DOI:** 10.1002/ece3.6517

**Published:** 2020-08-11

**Authors:** Aurore D. C. Panel, Ido Pen, Bart A. Pannebakker, Herman H. M. Helsen, Bregje Wertheim

**Affiliations:** ^1^ Groningen Institute for Evolutionary Life Sciences University of Groningen Groningen The Netherlands; ^2^ Laboratory of Genetics Wageningen University & Research Wageningen The Netherlands; ^3^ Field Crops Wageningen University & Research Randwijk The Netherlands

**Keywords:** *Drosophila suzukii*, fertility, life history, overwintering, reproduction, seasonal polyphenism, spotted‐wing drosophila, survival

## Abstract

Seasonal polyphenism in *Drosophila suzukii* manifests itself in two discrete adult morphotypes, the “winter morph” (WM) and the “summer morph” (SM). These morphotypes are known to differ in thermal stress tolerance, and they co‐occur during parts of the year. In this study, we aimed to estimate morph‐specific survival and fecundity in laboratory settings simulating field conditions. We specifically analyzed how WM and SM *D. suzukii* differed in mortality and reproduction during and after a period of cold exposure resembling winter and spring conditions in temperate climates. The median lifespan of *D. suzukii* varied around 5 months for the WM flies and around 7 months for the SM flies. WM flies showed higher survival during the cold‐exposure period compared with SM flies, and especially SM males suffered high mortality under these conditions. In contrast, SM flies had lower mortality rates than WM flies under spring‐like conditions. Intriguingly, reproductive status (virgin or mated) did not impact the fly survival, either during the cold exposure or during spring‐like conditions. Even though the reproductive potential of WM flies was greatly reduced compared with SM flies, both WM and SM females that had mated before the cold exposure were able to continuously produce viable offspring for 5 months under spring‐like conditions. Finally, the fertility of the overwintered WM males was almost zero, while the surviving SM males did not suffer reduced fertility. Combined with other studies on *D. suzukii* monitoring and overwintering behavior, these results suggest that overwintered flies of both morphotypes could live long enough to infest the first commercial crops of the season. The high mortality of SM males and the low fertility of WM males after prolonged cold exposure also highlight the necessity for females to store sperm over winter to be able to start reproducing early in the following spring.

## Graphical abstract

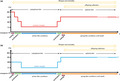

## 1 INTRODUCTION

Phenotypic plasticity can be defined as the ability of a given genotype to produce different phenotypes in response to environmental stimuli (Davidson, Jennions, & Nicotra, 2011; Gibert, Peronnet, & Schlötterer, 2007). It is considered an important mechanism by which organisms can cope with variable environmental conditions, where fitness‐maximizing trait values differ among these conditions. A continuous range of variation in phenotypes for a given genotype is called a reaction norm; when more discrete or clustered phenotypes are produced, the phenomenon is known as polyphenism (Kivelä, Välimäki, & Gotthard, 2013).

Polyphenism has been documented in many organisms, particularly in insects, where it is often highly adaptive. Indeed, the ability of insects to drastically alter their phenotype allows them to better cope with seasonal changes (seasonal polyphenism, e.g., dry or wet season forms in *Bicyclus anyana*), temporally heterogeneous environments (dispersal polyphenism, e.g., winged or wingless aphids), and varying population densities (density‐dependent polyphenism, e.g., solitary or gregarious locusts). It also enables them to partition labor among social groups (caste polyphenism, e.g., social insects; Simpson, Sword, & Lo, 2011). Very often, polyphenism is characterized by conspicuous morphological differences. In addition, the developmental program of polyphenic insects is fundamentally affected by the prevailing environmental conditions, which frequently leads to different life‐history strategies depending on the time of the year (Flatt, Amdam, Kirkwood, & Omholt, 2013). These life‐history strategies contribute differently to population growth and persistence. For instance, in some vinegar fly species, females can undergo a state of reproductive dormancy during cold months. This dormancy is associated with ovarian arrest and improved survival, which allows the vinegar fly populations to persist during winter. On the other hand, nondormant flies invest more in reproduction at the expense of survival, thus strongly contributing to population build‐up (Flatt et al., 2013). To better understand the population dynamics of polyphenic species, key life‐history traits need to be characterized for the different phenotypes.

Here, we focus on seasonal polyphenism of *Drosophila suzukii*, a multivoltine species that recently invaded Europe. This agricultural pest originates from South‐East Asia and threatens the worldwide fruit industry since it is highly polyphagous and infests a wide range of fruit crops as well as many wild host plants (Arnó, Solà, Riudavets, & Gabarra, 2016; Briem, Eben, Gross, & Vogt, 2016; Kenis et al., 2016; Lee et al., 2015; Panel et al., 2018; Poyet et al., 2015). The females use their serrated ovipositors to insert eggs in the flesh of ripening fruits. By feeding on the pulp, the larvae damage the fruits that then become unmarketable, causing significant economic losses to the fruit sector (De Ros, Anfora, Grassi, & Ioriatti, 2013; De Ros, Conci, Pantezzi, & Savini, 2015; Farnsworth et al., 2017; Goodhue, Bolda, Farnsworth, Williams, & Zalom, 2011). The flies can produce 5–15 generations per year, and their populations build up seasonally from low densities in winter and spring, until massive numbers over summer and autumn (Arnó et al., 2016; Panel et al., 2018; Wang et al., 2016).

In temperate climates, *D. suzukii* exhibits seasonal polyphenism in morphological, physiological, and behavioral traits, which enhances its survival under a range of stressful conditions (Enriquez, Renault, Charrier, & Colinet, 2018; Shearer et al., 2016; Wallingford & Loeb, 2016). This polyphenism manifests itself in two discrete morphotypes in the adult flies and is therefore essentially a type of biphenism. Juveniles developing in autumn at relatively low temperatures (10–15°C) and short photoperiod, emerge from the pupae as “winter morph” (WM) adults. This specific morphotype is characterized by a darker pigmentation and longer wings, compared with the “summer morphs” (SM) that emerge during summertime (Everman et al., 2018; Fraimout et al., 2018; Shearer et al., 2016; Stephens, Asplen, Hutchison, & Venette, 2015; Toxopeus, Jakobs, Ferguson, Gariepy, & Sinclair, 2016; Wallingford, Lee, & Loeb, 2016). The development into a specific seasonal form (WM or SM) is irreversible, and the morphotypes differ in gene expression and metabolic profiles, reflecting their different propensities for cold tolerance versus reproduction (Shearer et al., 2016). They may also differ in diet since fluctuations in resources between seasons likely imply different nutritional requirements and resource preferences (Rendon, Buser, Tait, Lee, & Walton, 2018; Rendon et al., 2019; Stockton, Brown, & Loeb, 2019). During the coldest months, *D. suzukii* adults, which are mostly WM, undergo a state of reversible reproductive dormancy (Mitsui, Beppu, & Kimura, 2010; Panel et al., 2018; Shearer et al., 2016; Toxopeus et al., 2016; Wallingford et al., 2016; Zerulla, Schmidt, Streitberger, Zebitz, & Zelger, 2015). As temperatures increase, the females resume egg maturation and oviposition (Grassi et al., 2018; Panel et al., 2018; Wallingford et al., 2016; Zerulla et al., 2015). Extensive monitoring programs in Europe indicate that WM flies arise from September onwards and can be found in substantial numbers until mid‐June whereas SM is caught from the end of May onwards and can persist until October (Panel et al., 2018; Vonlanthen & Kehrli, 2015).

Although the striking phenotypic differences between SM and WM of *D. suzukii* affect their individual performances under prevailing conditions, they may also have an impact on their seasonal population dynamics. Individuals of both seasonal morphotypes co‐occur in autumn and in spring (Leach, Stone, Van Timmeren, & Isaacs, 2019; Panel et al., 2018; Shearer et al., 2016), but they are expected to have different life‐history strategies, in particular regarding the investment in reproduction and survival. For instance, WM flies can better survive prolonged cold exposure and lower temperatures than SM flies (Shearer et al., 2016; Stephens et al., 2015; Stockton, Wallingford, & Loeb, 2018; Toxopeus et al., 2016). Furthermore, males are in general less cold‐tolerant than females, which results in a low availability of males to overwintering females in early spring (Shearer et al., 2016; Zerulla et al., 2015). Finally, cold exposure may cause reduced fertility in the few surviving overwintered males (Dalton et al., 2011; Grassi et al., 2018). To counteract the lack of males and their low fertility, overwintering females are thought to store sperm from autumn matings in their spermathecae during reproductive dormancy. As climatic conditions become more favorable for reproduction, they can resume oviposition without needing to mate again (Arnó et al., 2016; Grassi et al., 2018; Rossi‐Stacconi et al., 2016; Ryan, Emiljanowicz, Wilkinson, Kornya, & Newman, 2016). This reproductive strategy might affect the survival and the fecundity of the flies. For instance, prewinter matings could provide a nutritional advantage to overwintered protein‐deprived females through seminal fluids, leading to an increase in lifespan (Fricke, Bretman, & Chapman, 2010; Goenaga, Mensch, Fanara, & Hasson, 2012; Papanastasiou, Nakas, Carey, & Papadopoulos, 2013). On the other hand, sperm storage over long cold periods might have detrimental effects on the overwintering females’ fecundity due to a decrease in sperm quality and/or quantity (Novitski & Rush, 1949; Singh, Kochar, & Prasad, 2015). Moreover, reproduction can negatively affect survival of both males and females (Boulétreau‐Merle & Fouillet, 2002). In this respect, virgin *D. suzukii* adults might have a higher chance of successfully overwintering. Thus, the morphotypes and the sexes differ substantially from one another, both in various traits and in the challenges they encounter under natural conditions. This may also have led to the evolution of different life‐history strategies, such as the investment in current versus future reproduction, or in stress‐tolerance versus high reproductive performance.

Seasonal polyphenism in *D. suzukii* appears to predominantly affect their ability to overwinter and to cope with the postoverwintering conditions. However, the extent to which the two morphotypes differ in key life‐history traits during winter and spring is not well understood. These life‐history traits largely determine the population dynamics of this pest species, as natural enemies are essentially absent in newly invaded areas (Asplen et al., 2015; Miller et al., 2015), and its polyphagy ensures a near‐continuous availability of feeding and breeding resources (Kenis et al., 2016; Panel et al., 2018; Poyet et al., 2015; Stockton et al., 2019). Monitoring programs conducted in temperate countries show that overwintered females, which are mostly WM, resume reproduction as early as March. However, the explosive increase in population sizes usually occurs later and is observed from mid‐June onwards. This is also when SM males and females are rapidly increasing in abundance (Panel et al., 2018). Thus, implementing integrated pest management (IPM) strategies at the start of the yearly infestation cycle seems to be the best way to prevent the population to build up to massive numbers. In order to do this, we would first need to accurately predict population dynamics by estimating morph‐specific survival and reproduction, and their variations over time. Although a few population models have already been developed for *D. suzukii*, none of them takes these parameters into account (Wiman et al., 2014, 2016). This study aims to obtain detailed information on key life‐history traits of the two morphotypes under spring‐like conditions in order to parameterize future predictive models that could estimate the relative contributions of the morphotypes to population growth.

To compare how key life‐history traits in *D. suzukii* differ between the seasonal morphotypes, we studied their mortality and reproduction during and after a prolonged period of cold exposure. We experimentally explored the combined effects of (a) morphotype (SM vs. WM), (b) reproductive status (mated vs. virgin and precold mated vs. postcold mated) and (c) sex (female vs. male) on *D. suzukii* mortality and reproduction. In our experiment, we simulated an overwintering dormancy phase followed by early spring. Realistic temperature settings were used to accurately reflect environmental conditions and physiological states experienced by the pest in temperate regions. Our goal was to better understand the population dynamics of *D. suzukii* in early spring, a crucial period for population build‐up and initial host infestation.

## 2 MATERIALS AND METHODS

### 2.1 Fly stocks and culture

Adult *D. suzukii* were obtained from the laboratory‐reared population that had been started in 2013 from about 100 individuals, collected in the Gard region in south‐east France (GPS coordinates: 43.754059 N, 4.4595E). The flies were reared in plastic bottles (ca. 140 ml volume each) filled with a 30 ml rich cornmeal diet containing agar (10 g/L), glucose (30 g/L), sucrose (15 g/L), heat‐inactivated yeast (35 g/L, Mauripan), cornmeal (15 g/L), wheat germ (10 g/L), soya flour (10 g/L), molasses (30 g/L, Sweet Harvest Foods), propionic acid (5 ml/L), and Tegosept (2 g/L, Apex). The offspring of each generation was mixed and distributed over new bottles. Larval densities were standardized to approximately 100–150 larvae per bottle to avoid competition through overcrowding and to maintain the genetic diversity. The population was maintained in a climate‐controlled growth chamber set to 20°C, with a 16:8 L/D photoperiod to simulate summer conditions. All laboratory‐reared flies had thus a summer morphotype (SM). Relative humidity in the incubators was not strictly controlled, but was around 70 ± 10%.

### 2.2 Experimental design and measurements

**FIGURE 1:**
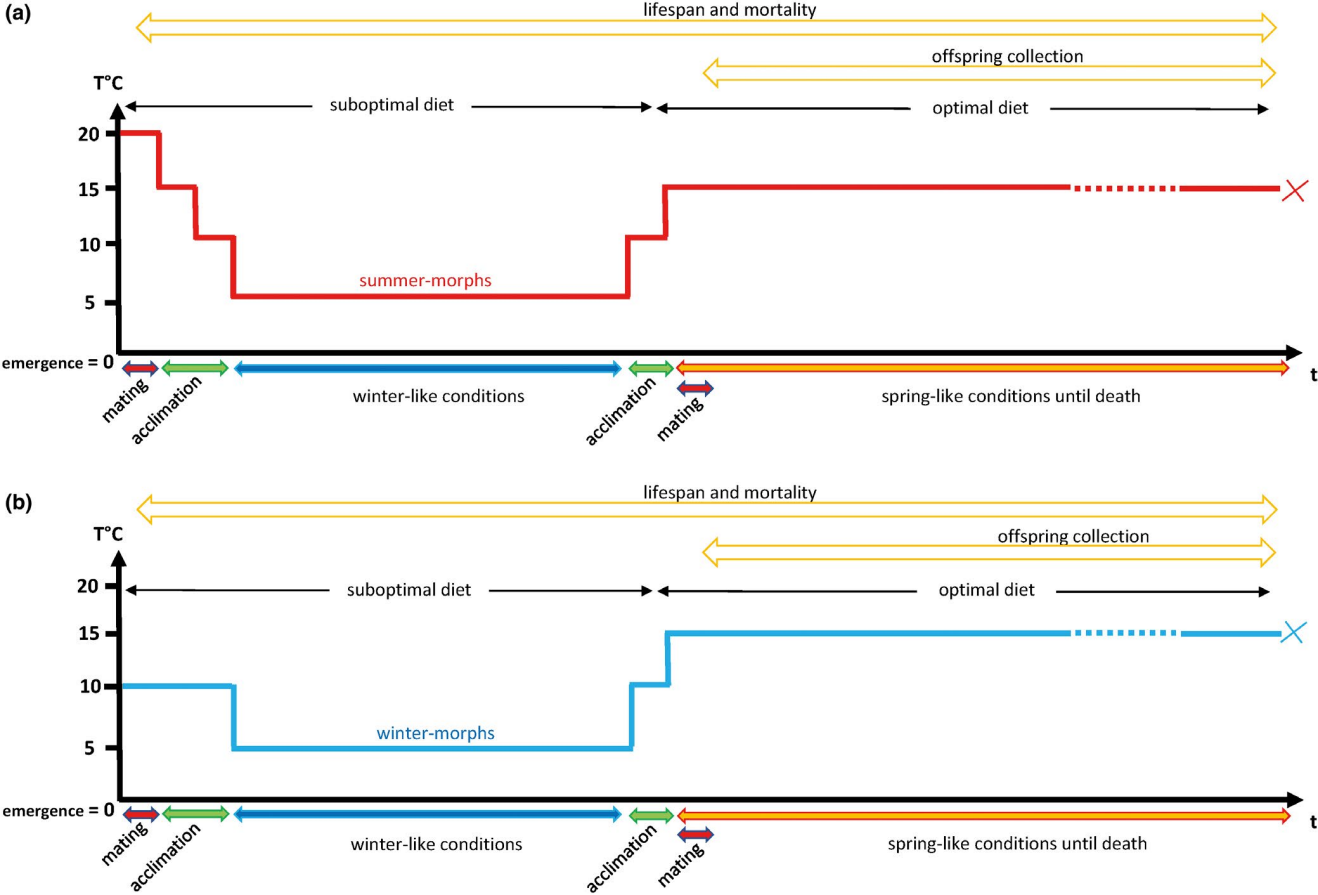
Graphical representation of the experimental setup. The timeline displays the treatments related to temperature, diet and reproductive status undergone by the experimental groups of *D. suzukii* summer‐morphs (a) and winter‐morphs (b). The flies were either kept virgin or mated before or after the cold‐exposure period (see Table 1). The life‐history traits that were assessed are also indicated

Firstly, SM and WM flies were induced by rearing larvae at different temperatures (SM and WM induction). Then, the experimental groups of emerged adults were preacclimated to cold conditions and subjected to a prolonged period of low temperature (31 days at 5°C) and suboptimal diet to simulate winter conditions and induce reproductive dormancy (cold‐exposure treatment). Survivors were subsequently given a progressive acclimation to warmer conditions, and from then on, maintained in spring‐like conditions (15°C) with an optimal diet until all of them had died (post‐cold exposure treatment; Figure 1). The following treatment groups were compared: “pre‐cold mated females,” “post‐cold mated females,” “virgin females,” “post‐cold mated males,” and “virgin males.” For each group, there were 20 vials with 5 individuals per vial, for a total of 100 individuals per treatment. This was done both for WM and SM flies (Table 1).

**TABLE 1:** The 10 experimental groups (*N* = 100 per group) subjected to 31 days of cold exposure (5°C) and subsequently maintained in spring‐like conditions (15°C) until death

Experimental groups	Life‐history traits	
	Mortality	Reproduction
Precold mated WM female	X	X
Precold mated SM female	X	X
Postcold mated WM female	X	X
Postcold mated SM female	X	X
Postcold mated WM male	X	X
Postcold mated SM male	X	X
Virgin WM female	X	
Virgin SM female	X	
Virgin WM male	X	
Virgin SM male	X	

Note

For each group, life‐history trait assessment is indicated by a cross (X). Some experimental groups of flies were mated before (precold) or after (postcold) the cold period, while the others were kept virgin. The assay was performed on *Drosophila suzukii* having either a winter morphotype (WM) or a summer morphotype (SM).

From emergence until death, all flies were checked for survival every 3–4 days while transferred into new vials. We measured their mortality during the prolonged period of cold exposure, and the lifespan of the survivors when maintained in spring‐like conditions. We also compared the reproductive output of WM and SM females that were maintained in spring‐like conditions after the cold exposure and that had mated either before or after this cold treatment (Figure 1). The females were all mated within a single four‐day period with standardized males that had not experienced cold exposure. The females had no further opportunities for mating during the entirety of the experiment. All vials that had contained mated females were incubated at 15°C and we collected the offspring until all the females had died (offspring collection). Finally, we tested the effect of the prolonged cold‐exposure period on the ability of SM and WM males to sire offspring. Following the prolonged cold treatment and the progressive acclimation to spring‐like conditions, SM and WM males were provided with virgin laboratory‐reared females for four days. These females had not been cold‐exposed, and their reproductive output was measured under spring‐like conditions every 3–4 days until they had died (Figure 1).

### 2.3 SM and WM induction

To generate flies for the experiment, groups of approximately 50 mature (10 days old) laboratory‐reared males and females were placed in 140 ml bottles containing the rich cornmeal diet and a scoop of yeast paste, and females were allowed to lay eggs during 24 hr at 20°C, 16:8 L/D photoperiod. Flies were then removed and bottles with eggs were randomly assigned to either 20°C, 16:8 L/D photoperiod or to 10°C, 12:12 L/D photoperiod to induce development of SM and WM individuals, respectively. The fly development from egg to adult emergence took approximatively 15 days at 20°C, and 60 days at 10°C. Within 2 hr upon emergence, virgin adults from both developmental conditions were collected, anesthetized on ice for a maximum of 1 or 2 min and distributed in same‐sex groups of 5 individuals in vials. These vials contained a sugar/yeast diet that is suboptimal for rearing *D. suzukii* (personal observation) and simulates food resource scarcity during the dormant period. It consisted of agar (17 g/L), sucrose (54 g/L), heat‐inactivated yeast (26 g/L, Mauripan), and nipagin (13 ml/L).

### 2.4 Cold‐exposure treatment

Newly emerged SM and WM flies were maintained in their respective incubators for 4 days. During this four‐day period, 20 female vials of each morphotype were provided with fresh laboratory‐reared males (2 males per vial of 5 females) to generate the “pre‐cold mated females” groups. The laboratory‐reared males were replaced in case they died to make sure the females had the opportunity to mate. The males were then retrieved and discarded. All other individuals were kept as virgins at this stage of the assay (80 vials with 5 females and 80 vials with 5 males for each morphotype). Then, all SM flies were subjected to a step‐wise acclimation process before the cold exposure treatment as follows: 15°C, 12:12 L/D photoperiod for 4 days followed by 10°C, 12:12 L/D photoperiod for 4 days. In the meantime, all WM flies were maintained at 10°C, 12:12 L/D photoperiod. All flies were then transferred to 5°C, 12:12 L/D photoperiod for 31 days. This temperature was selected because it allowed us to initiate reproductive dormancy in both SM and WM without strongly reducing survival (Rendon et al., 2018, 2019; Shearer et al., 2016; Tochen et al., 2014).

### 2.5 Postcold exposure treatment

After 31 days of cold exposure, the surviving flies were given a progressive acclimation to warmer conditions. They were incubated at 10°C, 12:12 L/D photoperiod for 4 days. Then, they were transferred to vials containing a rich cornmeal diet and incubated at 15°C, 16:8 L/D photoperiod for the remainder of their lives. The rich cornmeal diet mimicked the switch to alternative nutrient sources, such as pollen, nectar, and fruits, occurring in spring. The selected temperature aimed at simulating a temperature increase experienced after dormancy in temperate regions. To generate “post‐cold mated females” for each morphotype, 20 vials containing virgin females were provided with fresh laboratory‐reared males for 4 days from their first day at 15°C. Two males per vial were added, except when there was only one surviving female. In this case, one single male was added. The males were replaced in case they died to make sure the females had the opportunity to mate. They were then retrieved and discarded. Simultaneously, the “post‐cold mated males” groups were generated. For each morphotype, 20 vials containing virgin males were offered fresh virgin laboratory‐reared females for 4 days. The number of virgin females added in each vial was exactly the same as the number of surviving males.

### 2.6 Offspring collection

The offspring were collected every 3–4 days when they were approximately 2 days old (i.e., when the spots on the male wings were clearly visible). The sex and number of offspring per vial were determined under a stereomicroscope.

### 2.7 Statistical analysis

All data analyses were conducted using R version 3.6.2 (R Core Team 2019) in RStudio version 1.2.1114 (RStudio Team 2018). We used smoothing splines (“smoothers’’) or generalized additive mixed models (GAMMs) to fit age‐specific survival and reproduction, allowing for flexible smooth nonlinear relationships between age and the demographic parameters of interest. To correct for correlations between measurements obtained from flies in the same vials, vial was entered as a random effect in the models. The pammtools package version 0.1.17 (Bender, Groll, & Scheipl, 2018) was used to estimate age‐specific hazard (mortality) rates, while mgcv version 1.8‐31 (Pedersen, Miller, Simpson, & Ross, 2019; Wood, 2017) was used to estimate age‐specific fecundity, assuming a Poisson distribution for the number of offspring and a log‐link function. To speed up the calculations, with a small penalty in terms of accuracy, we used mgcv's bam() function, which is optimized for large datasets. The optimal number of basis functions or knots for the smoothing splines, which determines their “wiggliness,” was determined by the gam.check() function of the mgcv package. To judge the significance of age‐specific effects of categorical predictors like morphotype, we estimated separate smoothers for the different levels of the predictors and computed the differences between the smoothers and approximate 95% point‐wise confidence bands for the differences (Wood, 2017, p. 294). For age ranges where confidence bands do not overlap with zero, we say that there is a significant difference between predictor levels at those age ranges. Models with different sets of predictor variables were ranked according to the Akaike information criterion (AIC), which compromises between model goodness of fit and model complexity and is supposed to optimize out‐of‐sample predictive ability. Model weights were assigned according to the formula:

$$w_{i}=\frac{\exp\left(‐\frac{1}{2}\Delta {\text{AIC}}_{i}\right)}{\sum \exp\left(‐\frac{1}{2}\Delta {\text{AIC}}_{m}\right)}$$

Here, *w_i_* refers to the weight of model *i*, *M* refers to the set of all models under consideration, and ΔAIC is the difference in AIC between the focal model and the model with the lowest AIC score.

## 3 RESULTS

### 3.1 Lifespan and mortality

**FIGURE 2:**
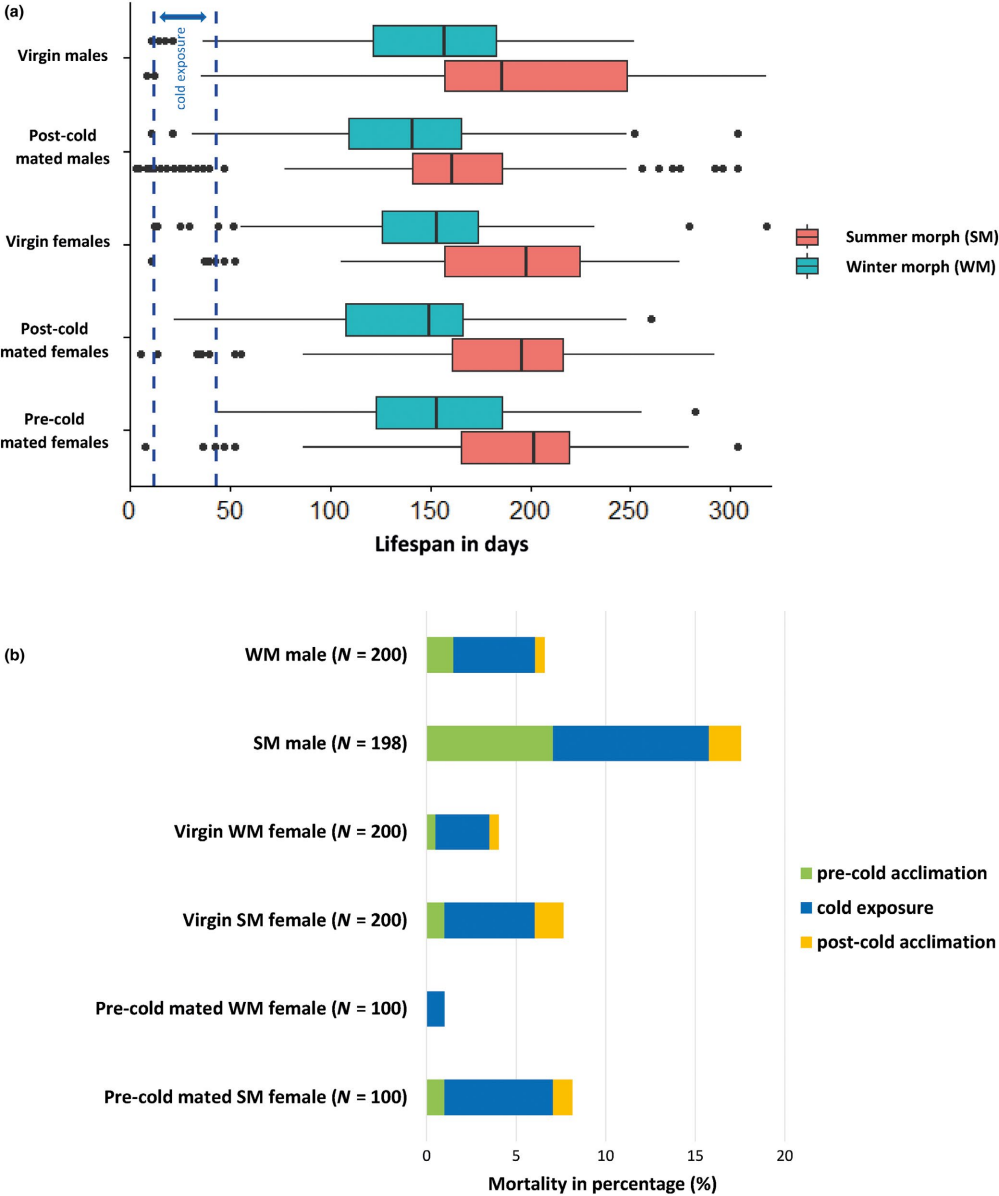
Lifespan and mortality of *D. suzukii* experimental groups (see Table 1). (a) Boxplots representing the lifespan of the flies (*N* = 100 flies per experimental group upon emergence). The flies were monitored throughout the experiment, from emergence (day 0) until death. The cold‐exposure period (31 days in total from day 12 until day 43) is delimited by the blue dashed lines. Each box represents the interquartile range of lifespan for the experimental group, the line in the middle is the median, the whiskers represent extreme values within 1.5 times the interquartile range, and the dots are the “outliers.’’ (b) Barplots representing the mortality of the flies during the acclimation and cold‐exposure periods. The percentage of flies dying (of those alive at the start of each period) during the 8‐day precold acclimation period, the 31‐day cold‐exposure period, and the 4‐day postcold acclimation period is shown for each group of flies. The number of living flies upon the first day of acclimation is indicated by the letter N

For the females, the median lifespans of the SM of different mating status ranged between 196 and 202 days, and between 149 and 153 days for the WM females (Figure 2a). The median lifespan of the males of different mating status ranged between 161 and 186 days for the SM, and between 141 and 157 days for the WM (Figure 2a). These observations indicated that, overall, median lifespan of *D. suzukii* varied around 5 months for the WM flies and around 7 months for the SM flies. The maximum lifespan we measured in our experiment was 318 days (around 11 months for a SM virgin male). Our GAMM analysis indicated that fly mortality over time was best predicted by including “morphotype,” “sex,” and their interactions as predictor variables in the model (Table 2). Adding “reproductive status” as predictor variable did not improve the model, indicating that the “virgin,” “pre‐cold mated” and “post‐cold mated” flies did not differ significantly in mortality under our experimental conditions.

**TABLE 2:** Comparison of GAMM models with different sets of predictor variables to predict age‐specific hazard (mortality) rates of *D. suzukii* experimental groups (see Table 1)

Rank	Predictor variables in each model	ΔAIC	Weight
1	Morphotype*Sex	0	1
2	Morphotype	12	0
3	Morphotype*Reproductive status	65	0
4	Morphotype*Sex*Reproductive status	78	0
5	Sex	117	0
6	Reproductive status	158	0
7	Sex* Reproductive status	184	0

Note

Candidate models were ranked from best (rank = 1) to worst fit (rank = 7) according to the Akaike information criterion (AIC). Model weights (Weight) reflect the probability that model *i* is the best model, given the data and the set of candidate models. ΔAIC is the difference in AIC between the focal model and the model with the lowest AIC score. All candidate models included “vial” as random effect. The predictor variables considered for this analysis were the morphotype (WM vs. SM), the sex (female vs. male), the reproductive status (mated vs. virgin and precold mated vs. postcold mated), and all possible interactions between these variables.

**FIGURE 3:**
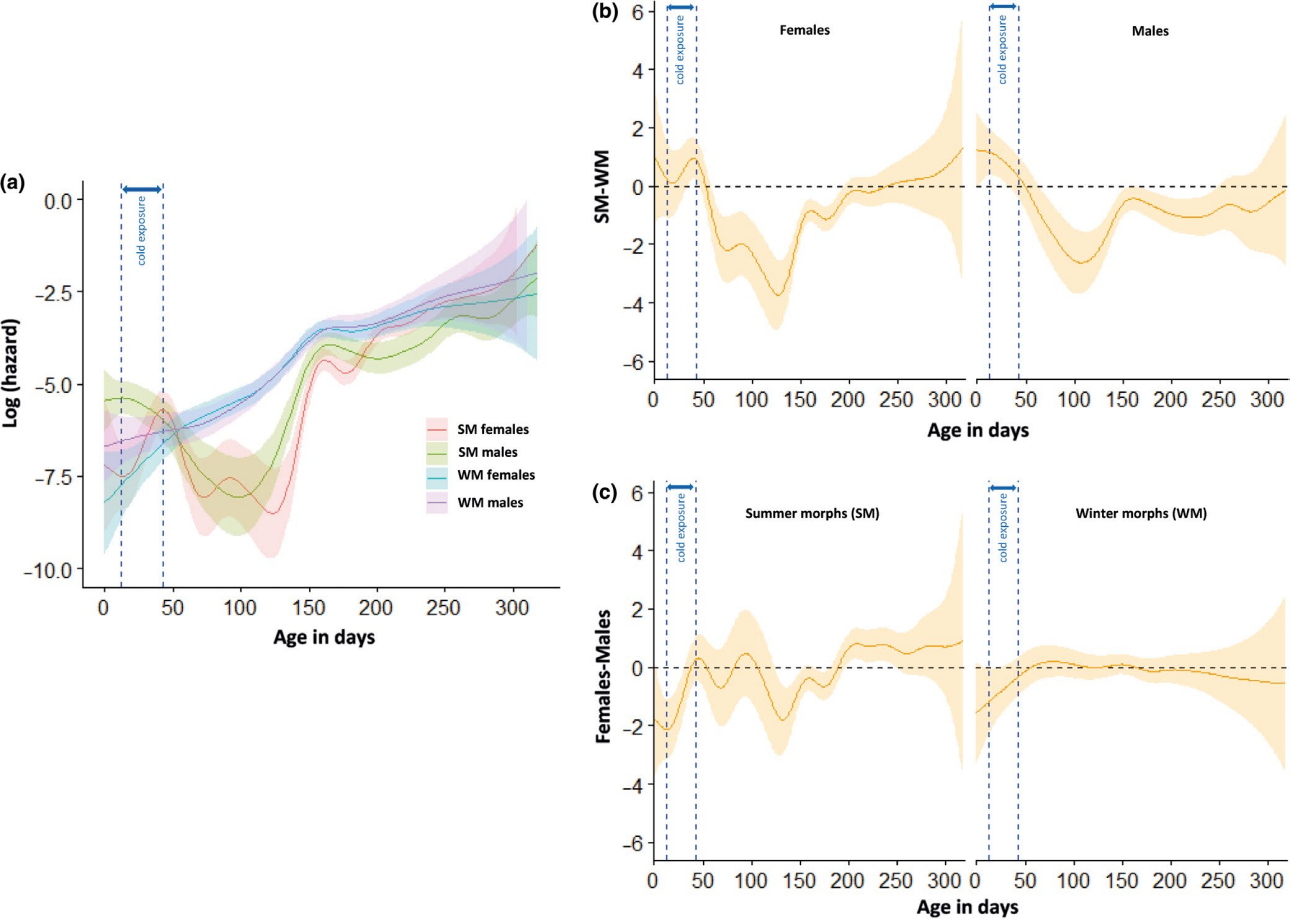
Age‐specific log hazard (mortality) rates of *D. suzukii* morphotypes. (a) Fitted smoothing splines of age‐specific log hazard (mortality) rates (solid lines), and 95% confidence bands (shaded regions) for the different levels of the selected predictors (here, the morphotype, the sex, and their interactions). (b) Difference between SM and WM fitted smoothing splines (see (A)) of age‐specific hazard (mortality) rates (solid lines), and 95% confidence band for each sex. (c) Difference between female and male fitted smoothing splines (see (a)) of age‐specific hazard (mortality) rates (solid lines), and 95% confidence band for each morphotype. For age ranges where the confidence bands do not overlap with zero, we consider that there is a significant difference in hazard between SM and WM, or females and males, at those age ranges

Mortality was generally low during the 31 days of cold exposure, with only 5% of the flies dying during the entire period. SM males had the highest mortality compared to the other groups of flies, with 7% of them dying during the 8‐day pre‐cold acclimation (when temperature was reduced stepwise from 20°C to 10°C), and another 9% dying during the 31‐day cold exposure at 5°C (Figure 2b). The GAMM analysis confirmed this observation and showed that, during the cold‐exposure period, SM males had significantly higher mortality rates than both sexes of the WM, and than SM females (Figure 3a–c). The mortality rate of SM females was increasing during the cold period and was significantly higher than that of the WM females during the latter part of this period (Figure 3a,b). WM males also appeared to have marginally higher mortality rates than WM females during the cold period (Figure 2b), but this difference was not significant in our GAMM analysis (Figure 3c). Under spring‐like conditions, WM flies (both males and females) had significantly higher mortality rates than SM, especially between days 50–150 (Figure 3a). No difference was found between the two sexes of the WM, whereas SM males and females slightly differed in mortality: SM males had significantly lower mortality rates than SM females between days 125–175, while they had higher mortality between days 200–250 (Figure 3a,c).

### 3.2 Female reproduction

**FIGURE 4:**
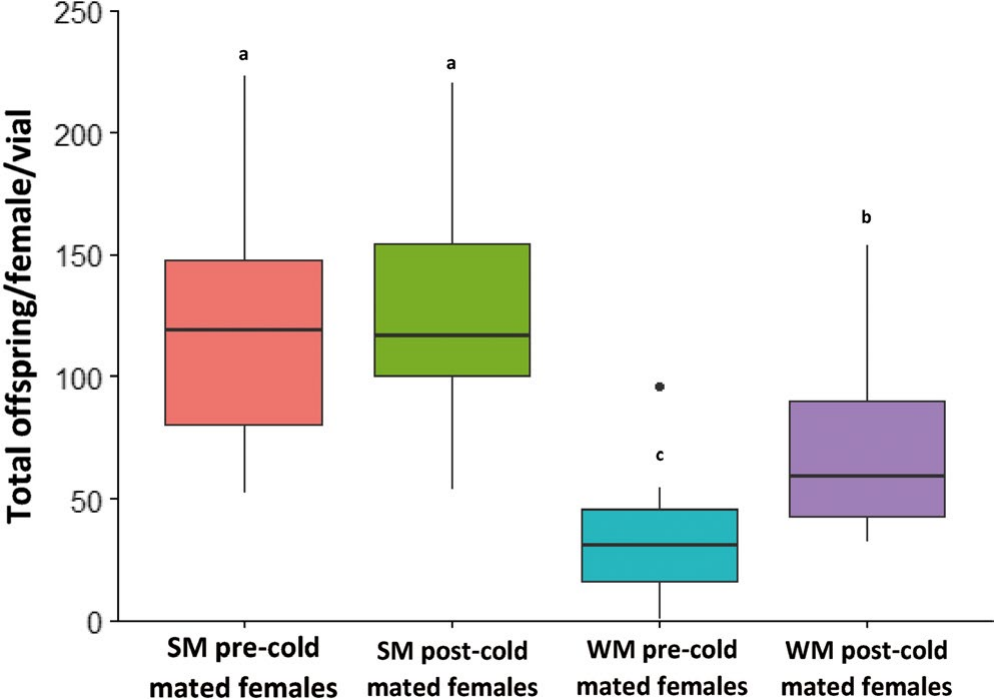
Lifetime reproductive output of *D. suzukii* females. The boxplots represent the total number of offspring produced per living female and per vial in the experimental groups of interest (see Table 1). Letters indicate statistical differences after Tukey's multiple comparison test (*p* < .05)

**FIGURE 5:**
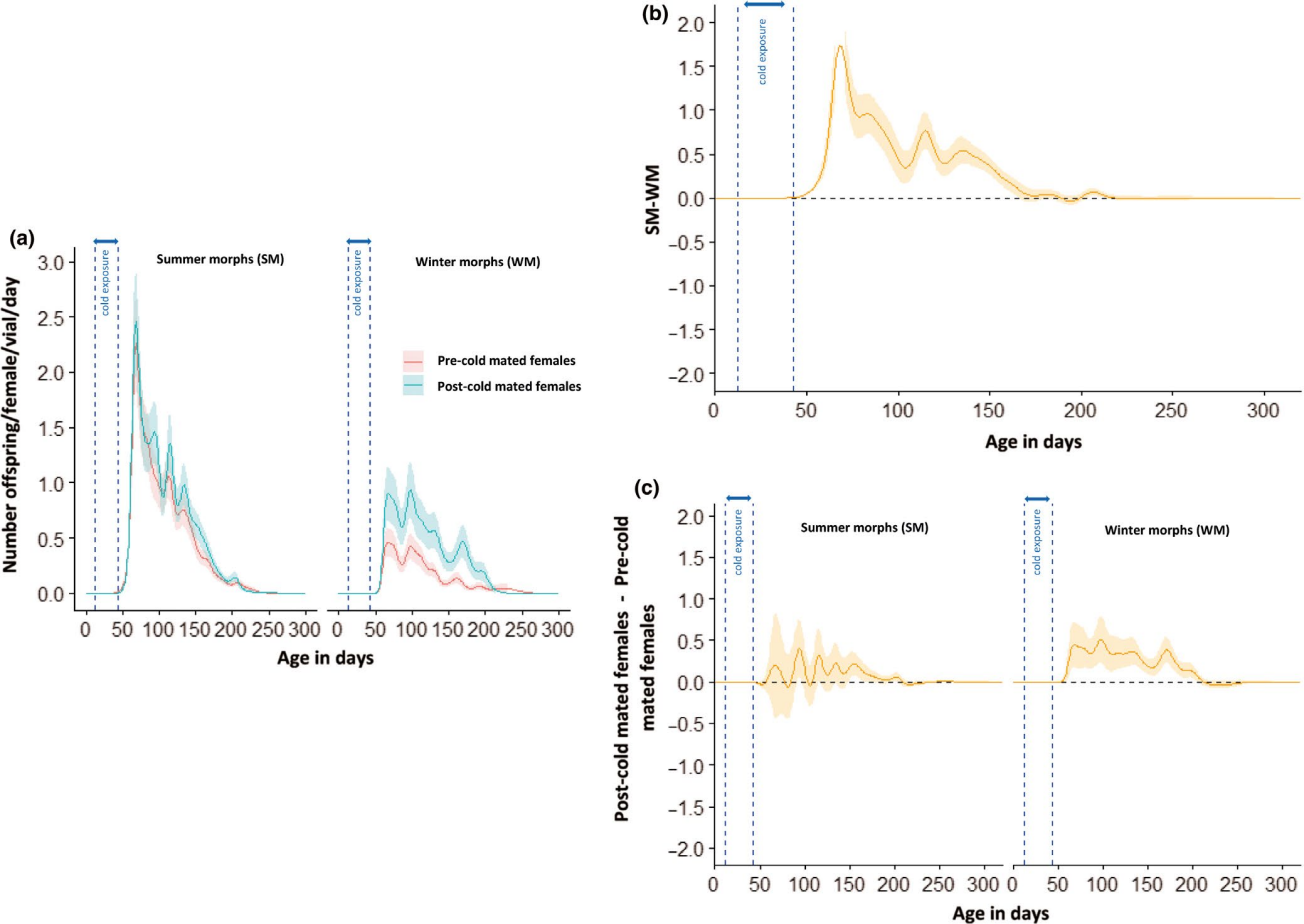
Age‐specific fecundity of WM and SM *D. suzukii* females that mated before or after the cold‐exposure period. (a) Fitted smoothing splines of age‐specific reproductive success of *D. suzukii* females for each morphotype (solid lines), and 95% confidence bands (shaded regions) for the different levels of the selected predictors (here, the morphotype, the moment of mating, and their interactions). (b) Difference between SM and WM fitted smoothing splines of age‐specific reproductive success (solid line), and 95% confidence band for this difference. For age ranges where the confidence band does not overlap with zero, there is a significant difference in reproductive success between SM and WM at those age ranges. (c) Difference between post‐cold and pre‐cold mated female fitted smoothing splines of age‐specific reproductive success (solid lines), and 95% confidence band for this difference and for each morphotype. For age ranges where the confidence band does not overlap with zero, there is a significant difference in reproductive success between postcold and precold mated females at those age ranges

The median lifetime reproductive success (expressed here as the total number of offspring produced per alive female and per vial) of the SM females that had mated prior to the cold exposure was 119.2 offspring, and 30.9 offspring for the WM females (Figure 4). Females that had mated after the cold exposure had a median lifetime reproductive success of 127 offspring for the SM females, compared to 59 for the WM females (Figure 4). This indicates that *D. suzukii* SM females had substantially higher lifetime reproductive success than WM females. Furthermore, whereas precold mating did not seem to diminish the lifetime reproductive success of SM females, it reduced the lifetime reproductive success of WM females by almost 50% when they were mated before the cold exposure (Figure 4). The GAMM analysis confirmed these observations and allowed us to get a better insight into the reproduction dynamics over the entire time course. Female reproduction was best predicted by including “morphotype,” “moment of mating” and their interactions as predictor variables in the model (Table 3). During the cold‐exposure period, neither the WM nor the SM females produced any offspring. Within a week from being under spring‐like conditions, the females started producing viable offspring. Irrespective of their moment of mating and their morphotype, females had the capacity to produce viable offspring for five months after the cold treatment (days 50–200). SM females produced significantly more offspring than WM females (an increase of up to twofold‐threefold; Figure 5a,b). Only for the WM females, there was an effect of moment of mating, whereby females that had mated after the cold‐exposure had significantly higher reproductive output than females that had mated before the cold treatment (Figure 5c).

**TABLE 3:** Comparison of GAMM models with different sets of predictor variables to predict age‐specific reproductive success of *D. suzukii* females subjected to different treatments (see Table 1)

Rank	Predictor variables in each model	ΔAIC	Weight
1	Morphotype*Moment of mating	0	1
2	Morphotype	119	0
3	Moment of mating	443	0

Note

Candidate models were ranked from best (rank = 1) to worst fit (rank = 7) according to the Akaike information criterion (AIC). Model weights (Weight) reflect the probability that model *i* is the best model, given the data and the set of candidate models. ΔAIC is the difference in AIC between the focal model and the model with the lowest AIC score. All candidate models included “vial” as random effect. The predictor variables considered for this analysis were as follows: the morphotype (WM vs. SM), the sex (female vs. male), the moment of mating (pre‐cold mated vs. post‐cold mated), and all possible interactions between these variables.

### 3.3 Male fertility

**FIGURE 6:**
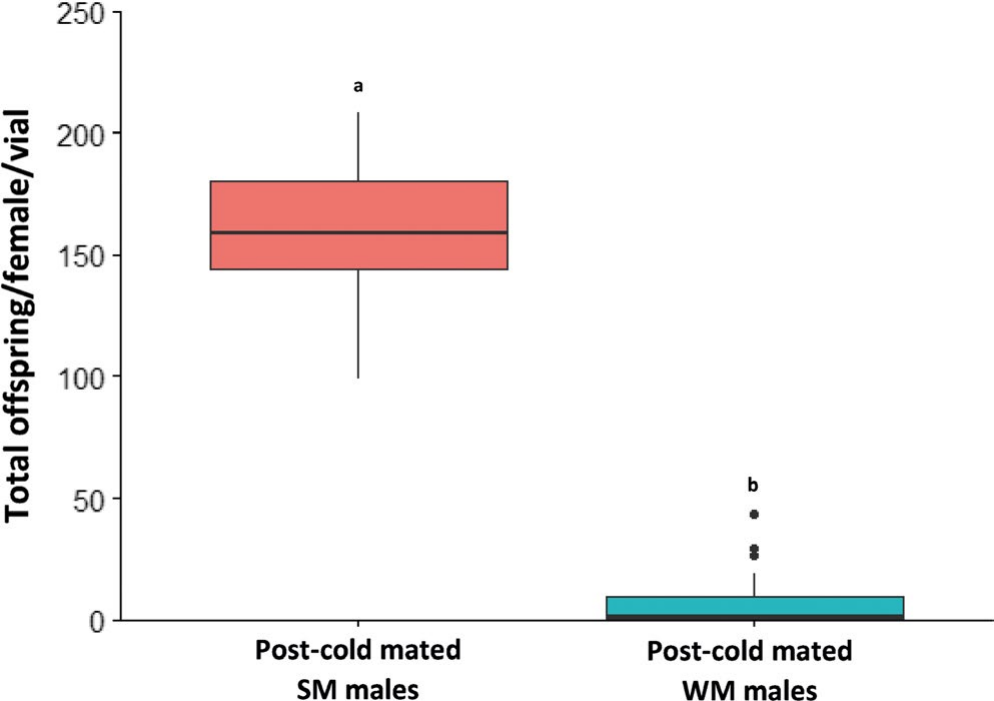
Fertility of *D. suzukii* males after cold exposure. After the cold‐exposure period, the WM and SM males were mated with SM females that had not been cold‐exposed, and we measured the lifetime reproductive output of these females. The boxplots represent the total number of offspring produced per vial and per living female. Letters indicate statistical differences (pairwise comparison, *p* < .05)

**FIGURE 7:**
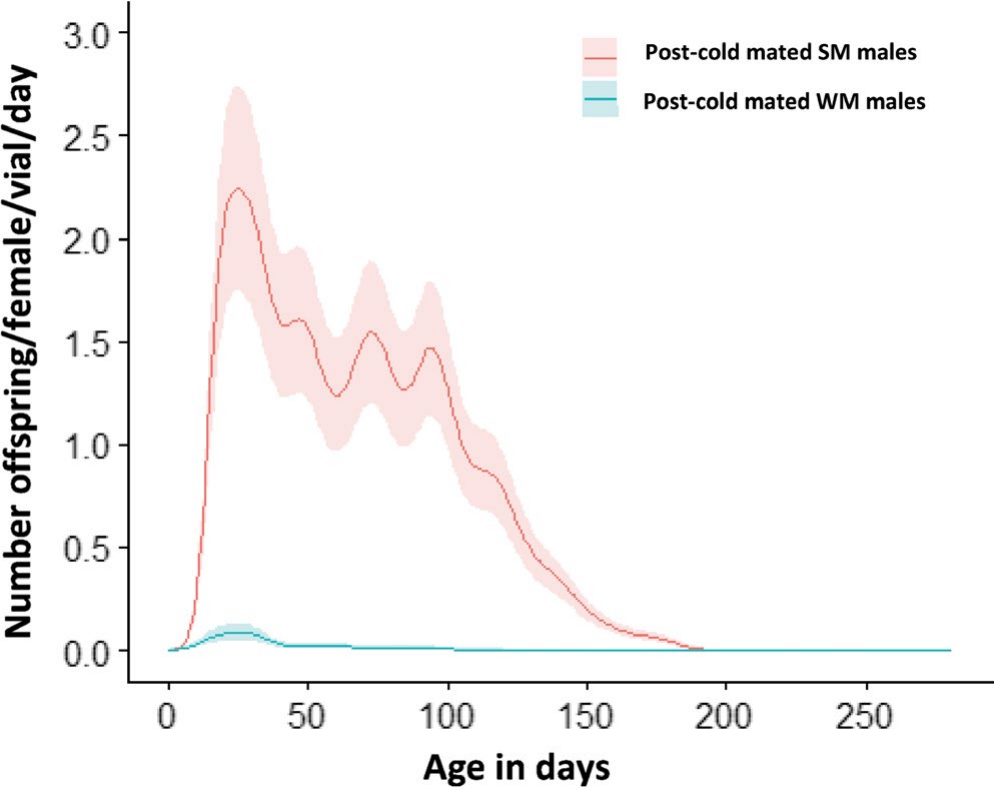
Age‐specific fertility of WM and SM *D. suzukii* males that underwent a period of cold exposure. The fitted smoothing splines of age‐specific fertility of *D. suzukii* males (solid lines), and 95% confidence bands (shaded regions) for the different levels of the selected predictors (here, the morphotype) are represented here. Male fertility is expressed as the reproductive success of the noncold‐exposed laboratory‐reared females that were mated with the experimental males (see Table 1) after the cold‐exposure period

The median fertility of the SM males (expressed here as the total number of offspring produced per alive females and per vial) was 175 offspring whereas it was 1 for the WM (Figure 6). These observations suggest that postcold mated WM males were almost sterile, whereas SM males had regained their fertility to levels comparable to noncold exposed males (not shown). The GAMM analysis confirmed this observation and revealed a significant difference between the numbers of offspring that females produced when they had mated with cold‐exposed WM and SM males. Virgin laboratory‐reared females sired by SM males were able to lay viable eggs over 200 days and produced significantly more offspring than the females mated with WM males (up to 20‐fold to 25‐fold; Figure 7).

## 4 DISCUSSION

In this study, we investigated key life‐history traits of the polyphenic insect pest *D. suzukii*. More specifically, we analyzed how *D. suzukii* seasonal morphotypes differed in mortality and reproduction during and after a prolonged period of cold exposure resembling winter and spring conditions in temperate climates. Better understanding the differences between these morphotypes during these periods is crucial to identify vulnerabilities of the pest that could be exploited in integrated pest management (IPM) strategies, and for anticipating the seasonal population dynamics. Our study reveals that median lifespan of acclimated *D. suzukii* maintained in spring‐like conditions after a simulated dormancy period ranged between 5 and 7 months for WM and SM, respectively. Even though the reproductive potential of WM flies was greatly reduced compared with SM flies, both WM and SM females that had mated before the cold exposure were able to continuously produce viable offspring for 5 months under spring‐like conditions. Mating before or after the cold‐exposure period substantially impacted the reproductive output of WM females, whereas this did not affect the reproductive performance of SM females. Interestingly, WM males were almost completely sterile after the prolonged cold exposure. This indicates important differences in the key life‐history traits of the two morphotypes under spring‐like conditions, which could be further analyzed to design morph‐specific IPM techniques against *D. suzukii*.

Our results on lifespan and mortality fit well with the findings of Rendon et al. (2019), who showed that overwintered flies maintained at 14°C on carbohydrate‐only diet have a median lifespan of around 4 months. The present study also confirms that *D. suzukii* females store sperm over winter, in agreement with previous observations (Grassi et al., 2018; Rossi‐Stacconi et al., 2016; Ryan et al., 2016). For the first time to our knowledge, we provide an estimate of the period during which overwintered females can produce viable offspring, relying on matings performed before dormancy. Taken together, these findings are consistent with the scenario proposed by us (Panel et al., 2018) according to which overwintered females live long enough after winter to infest the first commercial crops of the season and are the main source for the annual infestations. They also highlight the remarkable lifespan of these insects, and the vast number of offspring they can produce during their life, even when they only have access to mates for a single four‐day period.

We found that WM flies had a survival advantage during cold exposure compared with SM flies. This corroborates the conclusions of other assays investigating cold tolerance of both morphotypes (Rendon et al., 2018, 2019; Stockton et al., 2018). Indeed, although the SM flies had been gradually acclimated to cold conditions, likely enhancing their cold hardiness (Stockton et al., 2018), they were still more sensitive to cold exposure than WM flies. This observation shows that, even in relatively mild winter conditions, WM flies are better adapted than SM flies, which translates into lower mortality rates and longer lifespans. In contrast, under spring‐like conditions, the opposite pattern was observed with SM having lower mortality rates than WM. This differs from other studies that found that overwintered WM had higher survival rates than SM at all tested temperatures, both favorable and suboptimal (7, 9, 12, 15 and 17°C; Rendon et al., 2018, 2019). This may be due to differences in the experimental protocols compared with our colleagues. For instance, in our assay, flies of both morphotypes were gradually acclimated to both cold and spring‐like conditions, contrary to Rendon et al. who assigned overwintered WM and noncold acclimated SM to different diet and temperature treatments without transition temperatures. Furthermore, our WM were reared at 10°C whereas developmental temperatures used to induce the winter morphotype were higher (14 and 15°C) in other studies (Rendon et al., 2018, 2019; Stockton et al., 2018). Thermal responses of acclimated laboratory adult flies to heat and cold exposure, greatly depend on their developmental temperature (Hoffmann, 2010; Hoffmann, Sørensen, & Loeschcke, 2003; Schou, Loeschcke, & Kristensen, 2015). This suggests that a few degrees of temperature difference between our assay and other studies during fly development may have influenced thermal acclimation responses of WM in *D. suzukii*. These observations highlight the need for using ecologically relevant developmental temperatures during assays, resembling costs, and benefits experienced by natural populations undergoing daily and seasonal fluctuations. In that sense, comparing cold and heat tolerance of field‐captured WM with cold‐acclimated laboratory WM would be highly relevant to determine which rearing conditions induce WM flies that best reflect natural populations (Schou et al., 2015).

Interestingly, including reproductive status as a predictor of mortality did not improve the GAMM models, indicating that this physiological characteristic did not affect fly mortality to a discernible extent. For the cold‐exposure period, we performed an analysis of dynamic mortality patterns in females (see Figure S1) to test whether mating conferred a survival advantage to females during cold stress. Indeed, some *Drosophila* species can better tolerate desiccation and starvation in stressful environmental conditions when they are mated (Goenaga et al., 2012; Lacey Knowles, Brodie Hernandez, & Markow, 2005). This higher tolerance is thought to be acquired through copulation, when males transfer nutritive substances contained in seminal fluids to females. These nuptial gifts may help the females to face periods of food and water shortage (Goenaga et al., 2012). In our study, mated WM females had the lowest mortality during the cold‐exposure period, but compared with the overall low mortality of females during this period, there is no strong evidence of any such trend during cold stress. On the other hand, our results do not indicate a cost of mating for survival during cold exposure. These observations are consistent with the findings of Ryan et al. (2016) who showed that mating status did not influence the flies’ survival under cold conditions. Several studies highlighted that the effects of mating on vital demographic traits of females such as lifespan and fecundity may be quite variable since they depend on a variety of dynamic factors including nutritional status of females, age at mating, and strain. (Papanastasiou et al., 2013). Further investigation of these mechanisms is needed in controlled experimental setups to gain knowledge on *D. suzukii* reproductive strategies.

Under spring‐like conditions, we did not find evidence for a trade‐off between reproduction and lifespan either. There was no significant influence of reproductive status on female mortality. This may be caused by our experimental design, as the flies had ad libitum access to food and breeding sites. Also, virgin females may continue to produce and lay eggs, even when these are not fertilized (Narasimha et al., 2019; A.D.C. Panel, personal observation). Thus, our experimental setting could obscure any existing physical or energetic trade‐off based on limiting resources (Metcalf, 2016). Interestingly, though, we see a difference in the trade‐off between survival and reproduction between the two morphotypes. Whereas the age‐specific mortalities of females of the two morphotypes are very similar during the postreproductive period (from approximately day 200 onwards), they are very different during the reproductive period (from day 50–200). During reproductive age, WM females had substantially higher mortality than SM females, and, throughout life, their mortality rates fluctuated less than the mortality rates of SM females. Indeed, for SM females, the mortality decreased strongly during the reproductive period, both compared with the period of cold exposure and to the postreproductive period. This may reflect substantially different investment strategies in survival and reproduction for the two morphotypes.

Regarding the influence of sex on life‐history traits, we found that WM males and females did not differ in terms of survival, both under cold and spring‐like conditions. In contrast, SM males were significantly less cold‐tolerant than SM females during cold stress, resulting in higher mortality. These results are in agreement with previous studies on WM showing that above 1°C, there is no sex effect on survival during overwintering dormancy (Shearer et al., 2016). They also are in line with the findings of Dalton et al. (2011), who found that very few SM males survived 84‐day exposure to 10°C. Our data, combined with earlier studies, highlight that there are both sex‐specific differences in the conditions that are harmful to their survival, and that the threshold for this differs between the morphotypes (1°C cold exposure in WM, 5°C cold exposure in SM).

Our study clearly indicates a different investment in reproduction depending on the morphotype. We observed that WM females had a significantly lower reproductive output than SM, suggesting that cold tolerance might incur costs for reproduction. WM flies also typically have larger body sizes (Wallingford & Loeb, 2016). In the literature, cold tolerance and starvation resistance are indeed frequently related to larger body size and low fecundity in *Drosophila* (Cohet & David, 1978; Rion & Kawecki, 2007). More specifically, low temperature treatments result in altered transcript levels in *D. suzukii* which implies a redirection of energy from reproduction to winter stress tolerance (Enriquez & Colinet, 2019).

Intriguingly, we observed that overwintered WM males had almost entirely lost their fertility after 31 days of cold exposure. To verify this finding, we setup a small‐scale repeat experiment (data not shown) in which we regularly provided overwintered WM males (having undergone the same 31‐day cold treatment) with virgin females for prolonged periods. These laboratory‐reared females had not been cold‐exposed and were kept with the males under spring‐like conditions. In total, WM males (20 vials each with 5 males) were provided with two batches of 5 virgin females per vial, the first batch for one week and the second for three weeks. The reproductive output of the females was measured: no offspring emerged from a quarter of vials containing the first batch of virgin females and this increased to half of the vials for the second batch of females. Vials from which some offspring emerged contained very few flies, approximately 1%–10% of the number of individuals emerging from vials kept in parallel under the same spring conditions, but that had contained a batch of females mated with males from the standard culture. This confirms that WM male fertility after cold exposure was almost null and shows that the males did not recover their fertility over time.

The findings highlight the necessity for *D. suzukii* females to undergo sperm storage over winter and confirm the hypotheses of previous studies (Dalton et al., 2011; Grassi et al., 2018; Rossi‐Stacconi et al., 2016; Ryan et al., 2016). *Drosophila* males in general are often rendered sterile after a period of cold (Boulétreau‐Merle & Fouillet, 2002; Dalton et al., 2011; Giraldo‐Perez et al., 2016). However, our results show that, contrary to WM, overwintered SM males fully regained their fertility after the cold exposure period. This means that the morphotypes also differ in this key aspect of their life history. Disentangling the effect of temperature on male fertility and morphotype would require further experiments on both sperm production and sperm viability. To our knowledge, only one study has investigated male fertility of *D. suzukii* in field‐captured individuals, reporting that only few males contained sperm during winter (Grassi et al., 2018). However, the sperm viability of these males was not assessed. Investigating sperm viability of *D. suzukii* males of different morphotypes and exposed to various temperature conditions would be highly valuable for better predicting population build‐up and seasonal dynamics.

Our results highlight striking differences in key life‐history traits between WM and SM during winter and spring, which implies that the morphotypes contribute differently to the seasonal population dynamics. Population models should thus incorporate these differences in order to better forecast the pest population dynamics in spring and to design effective and efficient IPM techniques. Our data revealed highly dynamic patterns in hazards over time that differed largely between periods of cold exposure and postcold exposure. Therefore, the data presented were analyzed using an original approach, the GAMM, which allowed us to better understand the dynamics in mortality and reproduction over time for each experimental group. This analytical tool has already been used to model other aspects of *D. suzukii* biology (Leach, Van Timmeren, Wetzel, Isaacs, & Ross, 2019) and we strongly advocate its use in future studies in order to take temporal dynamics into account in survival analyses, and to allow a more detailed characterization of the differences in mortality or lifespan.

According to our findings, overwintered SM flies performed better than overwintered WM flies in terms of both survival and reproduction. Even if their survival performance might have been overestimated due to the experimental settings (mild winter conditions and prolonged spring conditions), we cannot exclude the possibility that, in nature, some SM flies might find refuge in shelters and successfully overwinter, as *D. melanogaster* also does (Boulétreau‐Merle & Fouillet, 2002). In the context of global warming, we can also expect that the percentage of SM flies surviving winter might increase in some geographic areas. Forecasting models combining the specific life‐history characteristics of both morphotypes, as described in this paper, and relevant climatic data, might be instrumental for predicting the *D. suzukii* populations outbreaks in the future (Langille, Arteca, & Newman, 2017).

Finally, our findings also have direct implications for current pest management strategies such as the sterile insect technique (SIT), the release of natural enemies, and the application of pesticides or bait sprays. In most cases, the success of all these density reduction methods is determined by the timing of the intervention, which is directly influenced by the biology and ecology of the pest. For example, SIT employs the release of sterilized males to compete with wild males. Considering *D. suzukii* females store sperm from autumn matings over winter, applying this approach in spring might not be as efficient as we would like. Especially since recent studies show that, when some species of polyandrous *Drosophila* females are exposed to cold conditions for a long period of time, the first male to mate a female fathers almost all of her offspring (Giraldo‐Perez et al., 2016). If this applies to *D. suzukii* females, releasing sterile males in autumn periods might be more effective. Similarly, biological control through the release of natural enemies also needs to be carefully timed, based on the pest life history. Furthermore, the efficiency of applying any pesticide or bait spray could differ between the two morphotypes, as they vary in many key life‐history traits that affect their (nutritional) requirements and behavior. To quantify this efficiency and its impact on the population growth, we have to take these differences into account. This study constitutes a first step towards achieving this objective and opens new avenues for research.

The data collected in this assay demonstrate that the developmental program of polyphenic insects such as *D. suzukii* can be fundamentally affected by the prevailing environmental conditions, which leads to different life‐history strategies depending on the time of the year. While the differences in temperature stress tolerance had been extensively studied before, our experiments indicate that the morphotypes also differ fundamentally in a range of key life‐history traits. The life‐history strategies that evolved for each morphotype, and the resulting trade‐offs, are likely tailored to the ecological conditions that impose challenges during the seasons. SM females and males are characterized by high reproductive output and low mortality, especially during the reproductive period. However, the SM flies, and especially the males, are more susceptible to cold than WM flies. They can capitalize fully on arising breeding opportunities and sustain these for several months, but they are vulnerable to cold stress. WM flies, in contrast, have a less fluctuating mortality rate throughout life, even under prolonged cold exposure, with a substantially lower reproductive output, even later on in life when conditions are more advantageous. They can cope well with extended periods of cold stress and a lack of mates after cold exposure, but at a cost to their reproductive potential. Gaining knowledge of these strategies will help making informed management decisions to reduce the seasonal population build‐up.

## CONFLICTS OF INTEREST

The authors declare no conflict of interest. The funders had no role in the design of the study; in the collection, analyses, or interpretation of data; in the writing of the manuscript, or in the decision to publish the results.

## AUTHOR CONTRIBUTION

**Aurore D. C. Panel:** Conceptualization (lead); Formal analysis (equal); Investigation (lead); Methodology (lead); Writing‐original draft (lead); Writing‐review & editing (equal). **Ido Pen:** Formal analysis (lead); Investigation (equal); Writing‐review & editing (equal). **Bart A. Pannebakker:** Conceptualization (equal); Writing‐review & editing (equal). **Herman H. M. Helsen:** Conceptualization (equal); Writing‐review & editing (equal). **Bregje Wertheim:** Conceptualization (equal); Formal analysis (equal); Investigation (equal); Methodology (equal); Writing‐review & editing (equal).

## ETHICAL APPROVAL

This study does not involve human participants or use of vertebrates. The use of insects does not require ethics approval.

## Supporting information

Figure S1

Figure S2

## Data Availability

All the data and R scripts used in this study are accessible in DataverseNL data repository. https://hdl.handle.net/10411/VFBTMK.
