# Supplementary figures and images for: Seasonal morphotypes of Drosophila suzukii differ in key life‐history traits during and after a prolonged period of cold exposure
Source: Ecol Evol. 2020 Aug 11;10(17):9085–99. doi: 10.1002/ece3.6517 (PMC7487234; doi:10.1002/ece3.6517)

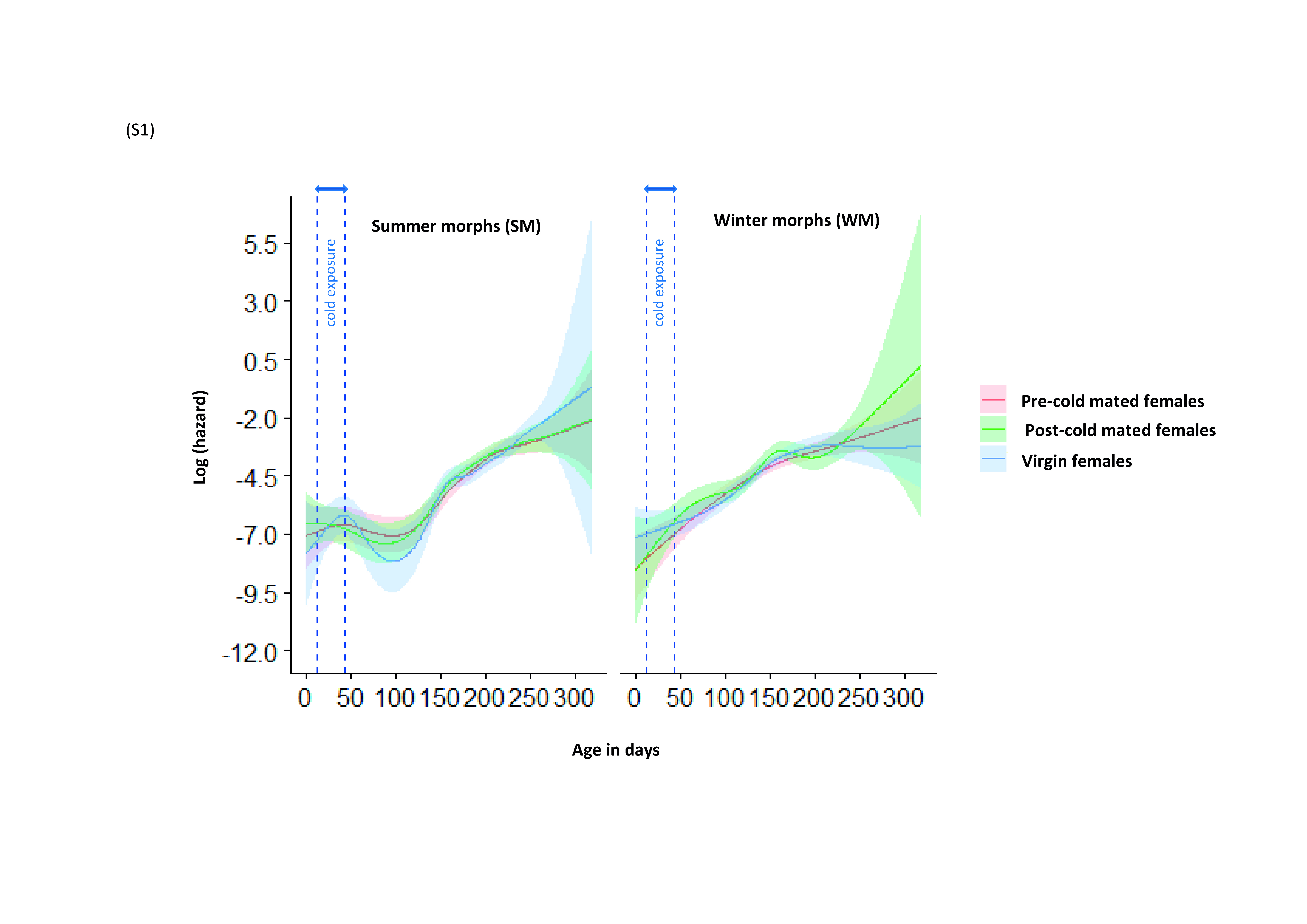

Supplement: Supplementary file 1 — Figure S1 [file ECE3-10-9085-s001.jpg]

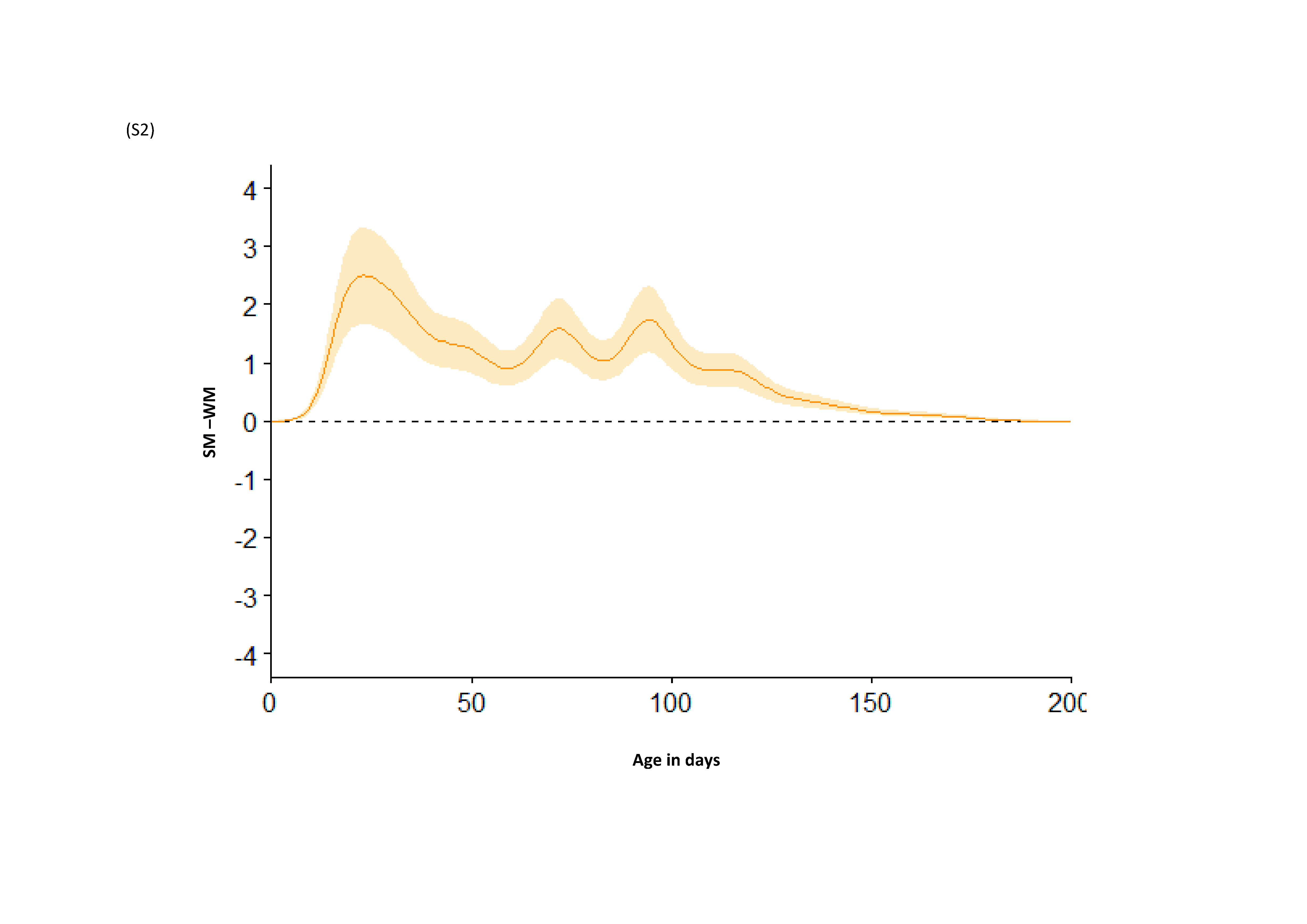

Supplement: Supplementary file 2 — Figure S2 [file ECE3-10-9085-s002.jpg]
